# An integrated perspective on transmutation of acute inflammation into chronic and the role of the microbiome

**DOI:** 10.25122/jml-2021-0375

**Published:** 2021

**Authors:** George Vithoulkas

**Affiliations:** 1. University of the Aegean, Syros, Greece; 2. Postgraduate Doctors’ Training Institute, Health Care Ministry of the Chuvash Republic, Cheboksary, Russian Federation

**Keywords:** Inflammation, microbiota, antibiotics, immune system

## Abstract

The Continuum theory and the Levels of Health theory were separately proposed to explain the myriad responses to treatment and understand the process of health and disease in an individual. In light of accumulating evidence on the intricate relationship between the human immune system and microbiome, an attempt is made in this article to connect these two theories to explain the transmutation of the efficiently responding immune system (through the acute inflammatory response and high fever) to one involved in a low-grade chronic inflammatory process (resulting in chronic disease). There is already enough evidence to demonstrate the role of the microbiome in all chronic inflammatory diseases. In this article, we discuss the mechanism by which subjecting a healthy person to continuous drug treatment for acute inflammatory conditions (at a certain time) leads to transmutation to chronic disease. Although this hypothesis requires further experimental evidence, it calls for a reconsideration of the manner in which we treat acute infectious diseases in the population.

## Introduction

In my paper, “The Continuum of a Unified Theory of Diseases” [1], the transmutation of acute disease to a chronic disease, signaling the “continuum” of disease states within an individual, was addressed, albeit grossly. This paper attempts to explain the likely mechanism of this transmutation further. It is with caution that the readers must review some of the conclusions in this study, which is based mostly on my experience of treating more than 150,000 cases.

In my book “Levels of Health” [2], I attempted to classify human health into 12 different “levels of health” statuses according to certain parameters. This paper attempts to connect the two ideas, the Levels of health and the Continuum theory, in light of inferences from immunological research. A paramount condition for this classification of levels is the response of the organism to acute infectious diseases. The ability of the organism to develop a fever high enough to “burn” the infectious agents is one of the main signs that the overall health of the individual is good [2, 3]. In this theoretical essay, 12 main categories of levels of health are described; however, these different levels of health and their subtle individualistic modifications on a global scale must actually result in hundreds of thousands (if not millions) of levels. As such, the level of health is essentially an individual attribute. As stated in the book, the Levels of health theory considers the grossest similarities shared by the individuals in the different groups from a homeopathic perspective.

The novel addition to this theory is that these levels are determined primarily but not exclusively by the degree of a harmonious or disharmonious symbiosis of microorganisms living in the gut or the skin. These are the archaea, bacteria, viruses, protists, fungi, and helminths that are the permanent inhabitants within the organism and are described as the human microbiome [4, 5]. Their balance and peaceful co-existence determine the degree of overall health status [6–8]. Indeed, this status depends on the critical number in and diversity of different colonies of microbes [9, 10]. 

Potentially, these microorganisms are of two kinds: useful microorganisms and harmful microorganisms [11]. When the colonies of useful bacteria or viruses are diminished, the colonies of harmful bacteria or viruses become enriched, and the inflammatory process begins [12]. This inflammation confirms that an internal war has been initiated in the environment of the microorganisms. It is initiated by the immune system when it senses that homeostasis is in danger, and the objective is to re-establish the lost balance.

### The Observational experience

I propose in this treatise that most chronic inflammatory diseases are due to transmuted microorganisms that have become toxic to the host. Purely monogenic diseases of Mendelian inheritance and noninflammatory conditions are excluded from this framework, as their mechanism differs from chronic inflammatory diseases [13].

### The construction of the human organism

In the books “Science of Homeopathy” [14] and “Levels of Health” [2], I have proposed the structure of the integral human organism. Understanding this construction becomes important to interpret the relative importance of organ systems in the body. Briefly, there is a hierarchy in the construction of the human being – some faculties/organs/organ systems are more vital for survival than others. These vital components are protected to a greater degree than the less important ones, even by the immune defense. This arrangement implies an effort on the part of the immune system to keep the disease disturbance as “superficial” as possible. An efficient immune system capable of easily adapting to disease-causing stimuli will not suffer at all but neutralize the agent effectively and proceed. With an increasing degree of compromise in efficiency, the immune system correspondingly allows serious infection. 

The hierarchy, in simple terms, on the physical level, follows the order: skin – mucous membranes and glands – muscular system – skeletal system – gastrointestinal system – renal system – lungs – liver and endocrine system – heart – the brain.

Over this structure, the pathological depth renders a layer of complexity to complete the picture. For example, although skin is more “superficial” than the mucous membranes and glands, the systemic pathology of autoimmune disease manifesting in the skin, viz., psoriasis, is a “deeper” disease than common tonsillitis. An invasive cancer of the bone is “deeper” than diabetic neuropathy. The reason this concept must be understood in this context is that the continuum theory and the current microbiome hypothesis speak of driving a disease “deeper” from “superficial”. It must be understood that these terms are relative and individualistic and not a generalized idea. In short, a deeper disease is one that has affected the more vital systems than a previously existing disease in that person.

### The battle

In the early years of life, the battle to maintain homeostasis in the face of pathogenic invasion usually starts as an acute infectious disease with high fever (e.g., tonsillitis, otitis, bronchitis, and enterocolitis), with the battleground being the mucus membranes and the glands [10, 15, 16]. If this superficial type of inflammation is suppressed by strong drugs that indiscriminately kill the fighting microorganisms, the result may be either recovery (unless the organism overcomes the side effects of the treatment) or an apparent elimination of symptoms but actual worsening of the microenvironment [17]. In the latter case, inflammation will proceed deeper and affect organ systems (such as the digestive, respiratory and nervous systems) or specific organs (such as the lungs, heart, liver, kidneys, and thyroid) [18]. If the imbalance at the microorganism level begins during infection and if the immune system of the host is not allowed to complete the battle on its terms due to the intervention of drugs, the overall health of the individual will be compromised, and the battle will be transferred to a deeper level in the form of sub-inflammation, which we recognize as a chronic disease [19, 20]. This battle, in the form of low-grade sub- inflammation, will continue for years unless the organism slowly restores the original/healthy composition of its microbiome [15]. This hypothesis implies that the correct way of treating such superficial infections is to allow the infection to run its course with minimal “support” from mild therapeutic means and not by chemicals that can kill useful microorganisms [21]. This approach, however, does not apply in emergency cases of severe inflammatory states where for instance, a septic condition is imminent. However, if the organism receives many drugs and the superficial inflammation is treated aggressively, the effect of the treatment will be suppressive instead of curative (a suppressive treatment is one in which drugs impede the process of natural recovery and do not allow the defense mechanism to execute the process of recovery in its own way and at its own pace). The defence mechanism, which is constantly striving to achieve the point of optimum function, when unable to deal with the infection in a curative way and simultaneously sensing the pressure from the action of drugs, will (by raising a second line of defence) transfer the battle to deeper organs to avoid a total collapse of the organism [22]. At this point, the inflammation ceases to be acute and turns to persistently low-grade [10, 20, 23]. Thus, death is prevented but at the cost of having the patient live with a chronic disease. Then, this transferred, deeper, sub-inflammation (a chronic condition now) will be much more difficult to treat [24, 25]. The progression of chronic disease will continue as long as the defense mechanism is unable to stop the increasing numbers of specific microorganisms that caused the imbalance. This fact is depicted in the results of different laboratory tests during the course of the chronic disease that demonstrates periodic exacerbations, indicating that there is a constant change of the microbiota according to the exacerbations and remissions [24].

It should not be misunderstood that the development of such chronic conditions is exclusively the result of specific suppression of acute infectious disease by drugs or of vaccination or exposure to any other toxic substance. It may also be the product of chronic severe stress or a psychological conflict that is deep enough that the organism cannot address it anymore. All such conditions can create changes in the composition of the microbiome, resulting in an increase in pathogen abundance or conversion of commensals to pathogens [12]. Examining the microbiota before and after vaccination would be interesting.

With the onset of such a conversion in the gut (from a commensal to pathogenic microbiome), a global battle begins between the different colonies of microorganisms—a war of life and death, for the survival of the host or of the pathogens! This is a typical battle for all patients suffering from chronic diseases [26], implying that all chronic diseases are maintained by different pathogens. A person’s life from that moment onwards depends on the outcome of this battle. Either the patient will recover by re-establishing balance, or their health will eventually become increasingly compromised until their final demise.

The transmutation from an acute infectious disease to a chronic one is occurring, among other phenomena, also because of the reduction in the abundance of useful microorganisms from the overuse of antibiotics or other chemicals that kill these bacteria that were maintaining the balance during health [4, 12, 27–29]. For example, when penicillin, a product of fungi, was discovered, its presence within the blood, especially whenever it is given in massive doses, kills bacteria, but eventually, the overuse of penicillin caused fungal diseases to increase in frequency and bacteria to develop resistance to the drug [12, 30–33]. In other words, if the augmented colonies of pathogens become established, they will keep neutralizing the beneficial colonies that are trying, under the authority and direction of the immune system, to re-establish the lost balance, i.e., homeostasis.

Therefore, it is obvious that a substratum connects the different microorganisms. This substratum is the environment, the nature of the constitution, or the predisposition of the individual organism. This substratum is not constant or steady but changes in accordance with the outcomes of these battles [12, 34, 35].

Furthermore, the microbiome also influences the psychology of a person [36]; for example, the bad psychological disposition of a patient suffering from even the simple flu or a common bacterial infection is well known [37–40]. During the course of the disease, all the changes in the symptomatology of a person, whether mental, emotional, or physical, coincide with changes in the composition of the microbiota [23, 41–43].

Next, we considered autoimmune diseases. Conventional medicine correctly defines a group of chronic diseases as autoimmune diseases, meaning (in effect) that the organism attacks itself, implying that the body’s defense mechanisms have gone awry. In reality, through unwise life behaviors and treatments, we have driven the organism to chaotic situations. It is well established today that many autoimmune diseases exhibit microbiome dysbiosis [15]. For example, multiple sclerosis patients or experimental autoimmune encephalomyelitis in mice express the respective T cell receptor for microbiota organisms [44]. 

Contrary to previous belief, it is currently recognized that chronic diseases are of a sub-inflammatory nature [45], probably maintained by certain types of mutated pathogens that were natural inhabitants of the gut in the past balanced state of the host [12]. We can thus infer that once a specific harmful virus, bacterium, or fungus has established supremacy, the health of the individual is severely compromised, while the balanced symbiosis within the microorganisms is lost.

### The education of the immune system

It is very important to consider that during the battle of the host with infecting agents, the immune system, whose objective is survival of the host, is actually simultaneously learning what to do in response to attack by a myriad of epidemic agents [46]. If this self-education of the young immune system (during childhood) is not allowed to complete its course with periodic high fevers and other inflammatory defenses, an increasing number of patients develop chronic diseases later in life [1, 15].

It is also worth remembering that an immune system that is not well-trained will become allergic to natural substances, such as the pollen of flowers, plants, pets, and foods, which are things that should bring joy to life rather than torment, as happens for children with allergies [47–51]. Allergic patients suffer not only physically but also mentally/emotionally from bad moods, anxiety, depression, phobias etc, exhibiting the connection between gut flora and the psychology of the patient [52–54]. It is interesting how the anxiety of patients with hypochondriasis often concerns their gut function. It is impressive how such patients themselves point out this connection to the doctor. This situation is the result of over-medicating; we have ended up with a great number of people suffering from allergic conditions in Western society [53]. Today, globally, the number of people affected by allergies is over 700 million, and approximately 40% of children are affected [55, 56]. These figures do not count adverse drug reactions and anaphylaxis, which are in themselves considerable. The burden is greater in the Western world, with over 7.8% of adults in the US suffering from hay fever and up to 40% of the population showing sensitization antibodies (namely, IgE) [55, 56]. However, the picture is different in the countries that more slowly adopted the use of drugs. In 2014, Kung *et al.* stated: “*Food allergy has been traditionally perceived as being rare in Africa. However, the prevalence of other allergic manifestations such as asthma and atopic dermatitis continue to rise in the higher income African countries”* [57]. It is also interesting that, until recently, neuromuscular diseases, such as multiple sclerosis, amyotrophic lateral sclerosis (ALS), and myasthenia gravis, were absent in the African continent, which did not have access to antibiotics and vaccinations [58].

This situation clearly demonstrates that, in recent decades, the deeper chronic diseases that have developed within Western populations are probably the result of the disturbance of the microbiota in these populations by excessive use of medications [59, 60]. In contrast, the economically challenged people of Africa, who did not have access to the said drugs, have been exempt from such disturbances. However, the incidence of allergies and neuromuscular diseases will rise in African populations as well [61, 62], as soon as they have access to the same drugs we use, due to the rise in their standard of living [63–66].

Allergies signify that humans are no longer fit to live in a natural environment, and as such, the environment appears to be inimical to these unfortunate people. While there may be other factors at work, such as pollution and bad nutrition, the fact remains that most of the population is not affected to the degree that the allergic patients are affected by the environment [67].

### Levels of health and the microbiota

The immune system maintains homeostasis not as a stagnant steady-state but as a dynamic equilibrium between slightly balanced and slightly unbalanced states [2, 68]. The highest level of health belongs to those organisms that maintain an excellent balance in their microbiome [68]. Moving down the levels from there, the immune system is increasingly compromised/weakened in its defense. At the lower levels (5 or 6), immune systems that are constantly fighting this battle are encountered, as they are constantly being attacked by pathogens trying to establish their colonies. This situation is clinically appreciated as repeated infections and severe infections [2, 69–71]. Here, although weaker than at higher levels, the immune system is still fighting to keep the microbiota balanced. However, descending further to levels 7, 8, and 9, altered microbial environments occur [4, 9, 15]. A state where pathogens have succeeded in their endeavor results in chronic inflammatory disease. When an organism has entered a chronic disease state, an overall shift in homeostasis occurs to survive in the new conditions created [72]. Afterward, a constant war prevails in the organism to maintain the optimum balance under the new circumstances and prevent the chronic condition from worsening. This phenomenon may be recognized clinically by periods of exacerbation and remission, which is characteristic of most chronic diseases [73–76]. Usually, in most cases, the beneficial microbiota constituents lose the battle for survival as the disease worsens and eventually involves other organs and systems, leading to the final demise of the patient [77].

The main characteristic of the first six levels of health, which separates them from the other six levels of increased chronic morbidity, is their possibility of raising a high fever in response to infectious agents 2. It must be noted here that the infectious agent is only the trigger; it is the instrument that activates the existing predispositions of the organism, as is expressed in the dormancy of the pathogens that, when triggered and awakened, start attacking the host [12, 78–80].

The Levels of Health Theory [2] explains that at the upper six levels, especially levels 1, 2, 3, and 4, the patient responds to an acute infectious disease by developing a high fever, counteracting the infectious agent. However, the infections in patients who are level 5 or 6 are more severe due to comorbidities that already exist at these levels. Medical assistance is usually required during these infections. From levels 7 to 12, deeper diseases, such as autoimmune conditions, neuromuscular diseases, ALS, active-stage multiple sclerosis, Alzheimer’s disease, dementia, chronic obstructive pulmonary disease (COPD), osteoarthritis, type II diabetes mellitus, systemic lupus erythematosus, psoriasis vulgaris, psoriatic arthritis, rheumatoid arthritis (RA), ulcerative colitis and heart diseases, are established. All these diseases have a progressive course starting from level 5 or 6, which is the beginning stage where the damage is not considerable and they are still amenable to treatment, progressing in morbidity through levels 7, 8, and 9 or even further when moving deeper to the last stages of health in levels 10, 11 and 12. At these levels, the organism is no longer able to develop a high fever (fever, if it develops, will be only mild) due to its increased morbidity [81]. Very high fever can develop at these levels if infected by highly virulent pathogens, and in such a case, the fever will prove fatal to the patient, as the immune system is already too weak to support the organism [82–84]. This phenomenon is seen in hospital infections and all chronic cases that are in their terminal stages; the patients suddenly develop a high fever that ends their lives. The mortality rate from these “final” fevers is very high [85]. Even if the patients do not die, as may happen if drugs manage to save their lives, they will still remain in a state such as dementia or complete exhaustion.

The difference in the pattern of fever during serious infections and their outcome has been reported by Bhavani *et al.*, indicating that there is indeed a difference in the way fever defense is generated at different levels [86]. In this context, the inference is that the cessation of the ability to generate a high fever in the presence of an infectious agent, which was possible in the past, indicates that the immune system is already compromised to a certain degree and that an active, chronic sub-inflammatory process is underway [1].

What we observe at these levels, for example, in RA, psoriasis vulgaris, or psoriatic arthritis in the beginning stages of their chronic disease, is that they still maintain the ability to develop a high fever during an infectious condition, such as viral or bacterial pneumonia. This can happen as long as the peripheral symptoms of inflammation in the joints or on the skin are present, but as soon as these symptoms have disappeared through suppression due to exposure to cortisone, methotrexate, or other biological agents, the whole impact of the disease enters another phase. This phase is a much deeper one in which the inflammation in the joints disappears to a great degree, but then, the central nervous system is affected with severe anxiety, panic attacks, depression, and lack of energy, together with the possible involvement of deep organic dysfunction in organs such as the heart, liver or kidneys. This is further confirmed by the phenomena that occur upon cessation of drug treatment in patients with RA who receive regular anti-inflammatory drug therapy and have remission of all symptoms. If the inflammation returns to full strength in the joints, the organism is able to develop a fever again in response to an acute infectious disease, while at the same time, all the deeper symptoms that existed during the suppression period, such as the decrease in energy, depression, panic attacks, and heart involvement, disappear. This process has been historically called a “syndrome shift” [87, 88], but actually, it is not a shift on the same level but rather to a deeper one.

Our inference from these clinical experiences is that, though admittedly strange, once the organism has entered into this deeper chronic inflammatory state, it seems to stop being affected by acute infectious diseases unless infected with a very virulent pathogen (e.g., nosocomial infections), in which case, the infection will lead to the demise of the patient. 

If a patient regularly acquires a yearly infection of some type, such as influenza, bronchitis, otitis, or cystitis, and suddenly ceases to acquire them for a few years, there is a possibility that a chronic condition has begun. We must investigate what has happened in this patient that stopped incurring infections with high fevers. This may indicate the beginning of an anxiety state, depression, malignant high blood pressure, the beginning of an autoimmune disease, or any other serious chronic disease condition. Such diseased individuals will remain unaffected by a virus that has infected the rest of the family [89]. It falsely appears that such an individual is “protected” from infection. In contrast, such an “immunological silence” indicates that the individual has entered a state with a seriously compromised immune system. The organism is busy dealing with a chronic sub-inflammatory condition, ignoring the infectious agent in the environment.

It has been observed that if a child has recurrent staphylococcal tonsillitis and antibiotics are prescribed successfully every time, the infection keeps returning until it ultimately manifests in the bronchi or the lungs, and the culture will now show *Proteus*, *Klebsiella*, or even worse, *Pseudomonas aeruginosa*, which are deeper, stronger antibiotic-resistant infections that are known to cause very serious infections [90]. This happens when the number of colonies of pathogens crosses a threshold [10]. Interestingly, pathogenic viruses or bacteria start increasing in abundance in the gut, and new infections are serious and difficult to cure [91]. However, this is when a transmutation occurs, and a chronic inflammatory disease starts manifesting [92]. This is the time when an organism passes from level 6 to level 7 or lower, where a chronic condition is established. This progression shows that the previously prescribed antibiotics have forced the immune system to alter the terrain of the intestinal flora and made the organism a fertile ground for, e.g., *Proteus* bacilli to develop unchecked. This situation is now much more difficult to suppress, even with a new generation of antimicrobial treatment.

However, it must be noted that the downward turn of health occurs not only after the overuse of antibiotics or medical drugs but also after exposure to any toxic substance or strong psychological stress capable of altering the microbiota [12].

### The terrain

While the process of mutation or transformation of commensal viruses and bacteria into infecting agents is well known to every physician, what is less known is that in most infection cases, the terrain plays the main role, creating the predisposition and the conducive environment for the infectious “trigger” to activate the dormant pathogenic viruses or microbes within the gut [15, 35, 68, 80]. The exact mechanism of the manifestation of a chronic disease is unknown, but it appears that the role of the composition of microorganisms is of primary importance [9, 15, 20, 27].

### The electromagnetic level

It is known that the basic building blocks of a human being are the force fields that constitute the primary level of our existence [93, 94]. The next level of fundamental building blocks of human beings is the microbiome, composed of trillions of microorganisms that have coevolved and live as commensals within the human body for mutual benefit [4, 95]. This highlights the potential damage to this level of the organism that can be caused by toxic substances.

If we consider that a constant effort exists to keep all types of microorganisms in a state of peaceful co-existence (in symbiosis), we see that there is a constant battle between the forces of life and the forces of destruction and death. This battle is nowhere more obvious than in the gut flora. If the immune environment changes, the ground and the terrain become conducive for some of the pathogenic viruses, bacteria, or fungi to multiply and overwhelm the organism, preparing the conditions for the death of the host [20, 27].

In conclusion, since we do not yet know the exact role of each virus or bacterium, we should only very carefully interfere with the gut flora. Therefore, an ideal way to neutralize a pathogen during an acute infection or under stress is not through drugs that kill the pathogens directly but by changing the environment in which they thrive. Such a change can be manifest only by therapeutic modalities that use subtle energy remedies that directly affect the fields of forces of the organism, such as homeopathy. Homeopathy maintains that useful information is imparted to the sick organism, probably on an electromagnetic level [96], through the potentized homeopathic remedy that carries the information needed by the organism to recover. Of course, this therapy requires a highly trained practitioner.

The overall intelligence of the organism constituting the specific fields of forces governs the function of the organism. This aspect is addressed in homeopathy with the generic name of vital force [97]. These fields of forces within the organism retain all the necessary information and direct the optimal functioning of the human organism.

This understanding will demonstrate the usefulness of homeopathy to the medical profession, which addresses the disease at the most basic level of its existence—the vital force of the organism. Homeopathy deals with tangible and reproducible clinical effects [98–107]. It is true that the objection for its use, viz., no demonstrable material in the medicine, remains to be answered [108]. However, there is enough evidence to spur research that will help explain the science. It must be remembered that Max Planck explained the quantum nature of light, although it shook his classical sense of physics. The theory made practical sense, although it was without a theoretical basis at that time. The facts were explained by a paradigm shift that occurred much later with the advent of quantum physics [109]. Similarly, in homeopathy, we see a great deal of evidence accumulating, but it is being targeted for its inability to explain the mechanism of action in the realms of material science [108]. However, it has been recognized in astrophysics that the building blocks of life are fields of forces that constitute a complex electromagnetic field on which rests the inflow of energy to every living creature [93, 110, 111]. This is why homeopathy is so effective because it goes deeper and beyond the microbiota and affects the force fields of the organism, which are the primary building blocks of life [112–116]. If this electromagnetic environment of the organism is positively affected through the information contained in the potentized homeopathic remedy, the electromagnetic field of the microbiome is rebalanced [96]. The rebalanced environment becomes unsuitable for pathogens to survive, and healthy conditions are restored. I propose that experimentation on these lines of thought be designed to try to explain the considerable clinical evidence for the effect of homeopathy. The rebalanced environment becomes unsuitable for pathogens to survive, and healthy conditions are restored. I propose that experimentation on these lines of thought be designed to try to explain the considerable clinical evidence for the effect of homeopathy [117–120]. 

There has been a tremendous increase in recent years in the incidence of chronic degenerative diseases. According to Zhongming *et al.*, [121], the reason for this increase has not been investigated properly thus far. An effort has been made in this paper to elucidate this problem. There is strong evidence that there is a correlation between the functioning of the healthy immune system and the condition of the gut microbiota [122, 123]. One common example is the presence of numerous bacterial colonies in the intestinal tract of humans. They seem to be in a delicate balance as long as the organism, as a whole, is in its highest health state.

## Conclusion

In this paper, we showed that there is evidence that treatment with antibiotics and corticosteroids has, in certain cases, a negative impact on the microbiota, which may be lasting and contribute to the emergence of a chronic degenerative condition. As a result, anomalies at this level seem to define the stage of chronic disease in their multiple chronic manifestations, which is responsible, to a large extent, for the status of health and disease. Each influences the other, and factors that affect one cause changes in the other. With this background, it seems plausible that the efficiency with which the immune system develops an effective inflammatory response to pathogens and maintains health depends to a great degree on the condition of the microbiota. If chemicals damage this first line of defense, other stressors that affect the microbiota may lead to low-grade chronic inflammation triggering a chronic degenerative disease to which the individual has a predisposition.

A healthy organism is able to raise a fever and start an inflammation process whenever a virulent pathogen enters the organism. Such inflammation should be treated with great care and sensitivity to avoid destroying the mechanisms existing in the microbiota that allow the organism to successfully fight acute infectious diseases.

Alternative ways of treating acute inflammatory conditions, especially homeopathy, could be investigated and tried as the first line of defense in treating such acute diseases before resorting to antibiotics or corticosteroids. Homeopathic remedies acting on the human energy field bring about an instant balance, leading to the re-establishment of a healthy microbiota and hence returning the organism to a state of efficient defense. Such evidence available today in the medical literature, links to the Levels of Health theory and the Continuum theory. We hope that this small contribution serves as a trigger for investigating this issue within medical research centers. It is evident and understandable that such an avant-garde theory needs further experimental confirmation and basic research to establish the parameters – e.g., through immune profiling before and after acute infection, which could predict who is sensitive and in danger of developing a chronic disease if their acute inflammatory process is interfered with or suppressed with chemical drugs, steroids or antibiotics. This article also aims to establish that the early suppression of fever is not always a wise practice, especially in children, which is well known and practiced amongst most pediatricians. The main limitation of the theory mentioned above is insufficiently robust research, confirmed by randomized, controlled, double-blinded studies to further support this hypothesis – definitely, the statistical (data) limitation is the main one. This has to become an immediate priority for this field of research. Of course, there is not so much research in this scientific area at all. My opinion is that, since some preliminary data already exist, properly designed clinical trials in the near future will be able to confirm the correctness of all the above.

## Acknowledgments

### Conflict of interest

The authors declare that there is no conflict of interest.

### Personal thanks

I am indebted to Dr. Seema Mahesh and Dr. Dionysios Tsintzas for their contributions to this paper.

### Authorship

GV conceived the idea, wrote the manuscript and obtained the relevant references, and is the sole guarantor of this work.
